# Beyond Borders: Monkeypox Case on Madeira Island

**DOI:** 10.7759/cureus.50715

**Published:** 2023-12-18

**Authors:** Filipa M Andrade, Guilherme N Faria, Maria L Ramos, Susana Franco, Tiago Sardinha, Sara Gouveia

**Affiliations:** 1 Family Medicine, Centro de Saúde do Bom Jesus, Serviço de Saúde da Região Autónoma da Madeira (SESARAM), Funchal, PRT; 2 Family Medicine, Centro de Saúde do Porto da Cruz, Serviço de Saúde da Região Autónoma da Madeira (SESARAM), Funchal, PRT; 3 Family Medicine, Centro de Saúde Dr. Rui Adriano de Freitas, Serviço de Saúde da Região Autónoma da Madeira (SESARAM), Funchal, PRT; 4 Family Medicine, Centro de Saúde Faial, Serviço de Saúde da Região Autónoma da Madeira (SESARAM), Funchal, PRT; 5 Family Medicine, Centro de Saúde do Caniço, Serviço de Saúde da Região Autónoma da Madeira (SESARAM), Caniço, PRT

**Keywords:** case report, primary health care, diagnosis, zoonosis, emerging infectious diseases, monkeypox

## Abstract

Human infection with monkeypox virus (MPXV) is characterized by a pox-like rash in various areas, including the anogenital region, and is accompanied by fever, headaches, fatigue, myalgias, and lymphadenopathy. These symptoms may occur a few days before the rash or simultaneously. Nonspecific and vague symptoms, particularly in the recent outbreak of the MPXV, have led to unrecognized or very mild prodromes, which can delay diagnosis. Diagnosis involves laboratory confirmation through polymerase chain reaction (PCR). The symptomatology of MPX is self-limiting, resolving in about two to four weeks. Therefore, the therapeutic approach includes supportive care, monitoring, intervention for possible complications (e.g., bacterial superinfection, cellulitis, and bronchopneumonia), and the implementation of preventive contact measures. This clinical case emphasizes the importance of conducting a thorough medical history and maintaining a high level of clinical suspicion, even in the absence of a history of contact with suspected or confirmed cases and in regions without active or suspected infectious cases.

## Introduction

Monkeypox, caused by the monkeypox virus (MPXV) within the Orthopoxvirus family, is a rare zoonotic disease similar to smallpox [1]. The virus is primarily transmitted to humans through direct contact with infected animals, especially rodents, which serve as reservoirs for the virus. Human-to-human transmission is possible but less common, typically occurring through close contact with respiratory secretions, skin lesions, or fomites [2]. In the 2022 MPXV outbreak, most confirmed cases with travel history reported travel to countries in Europe and North America [3]. The predominant mode of transmission is through sexual intercourse, particularly among men who have sex with men, often presenting with genital lesions [4]. The symptoms of monkeypox typically manifest within a range of 5-14 days after exposure, although onset can extend up to 21 days [5]. Typically, one to two weeks after infection, initial symptoms include fever, headache, muscle aches, and fatigue, followed by distinctive pox-like skin lesions that develop, progressing from macules to papules and vesicles filled with clear fluid, eventually resulting in scabs. In some cases, the rash starts with vaginal and perianal lesions [2]. Prompt diagnosis and isolation of suspected cases are essential to prevent further transmission. Laboratory testing is crucial for confirming MPXV, as it can be challenging to differentiate from other viral illnesses like chickenpox or smallpox [6]. Recent research has advanced our understanding of MPXV, aiding in developing diagnostic tools and potential vaccines. However, there is no specific antiviral treatment, and supportive care remains the mainstay of therapy [2,7]. This case highlights the importance of a comprehensive medical history and sustained clinical vigilance, even in the absence of contact with confirmed cases or in regions without active infectious cases.

## Case presentation

A 44-year-old male, born and residing in Madeira Island, Portugal, with a history of a brain tumor surgically treated in 2008, presented to the ED. He was not on regular medication and had no drug allergies. He had a history of childhood chickenpox. He presented at a non-scheduled health center appointment with complaints of a generalized, pruritic skin rash that began on the face and progressed to the trunk and limbs over the past week and a half. Additionally, he reported experiencing sweating, myalgia, and headaches for the last five days. He also had a sore throat for the past two days, which improved with antibiotic treatment prescribed over the phone by a friend. He denied having gastrointestinal or urinary complaints, asthenia, genital lesions, or any other symptoms.

His travel records indicated a vacation taken in Crete, Greece, about one month prior to the appointment, with two layovers (Orly and Lisbon). Throughout the duration of the stay, multiple occasions featured significant gatherings of people. The individual was not aware of contact with suspected or confirmed cases of MPXV infection or anyone displaying suggestive symptoms in the last 21 days.

In the last three months, he reported having had only one female sexual partner, with the most recent sexual activity occurring over a month ago. He denied any diagnosis of sexually transmitted infections in the last three months. He also denied contact with domestic or wild animals, group sexual activities involving object sharing in the last 21 days, or visits to saunas, Turkish baths, or similar establishments.

**Figure 1:**
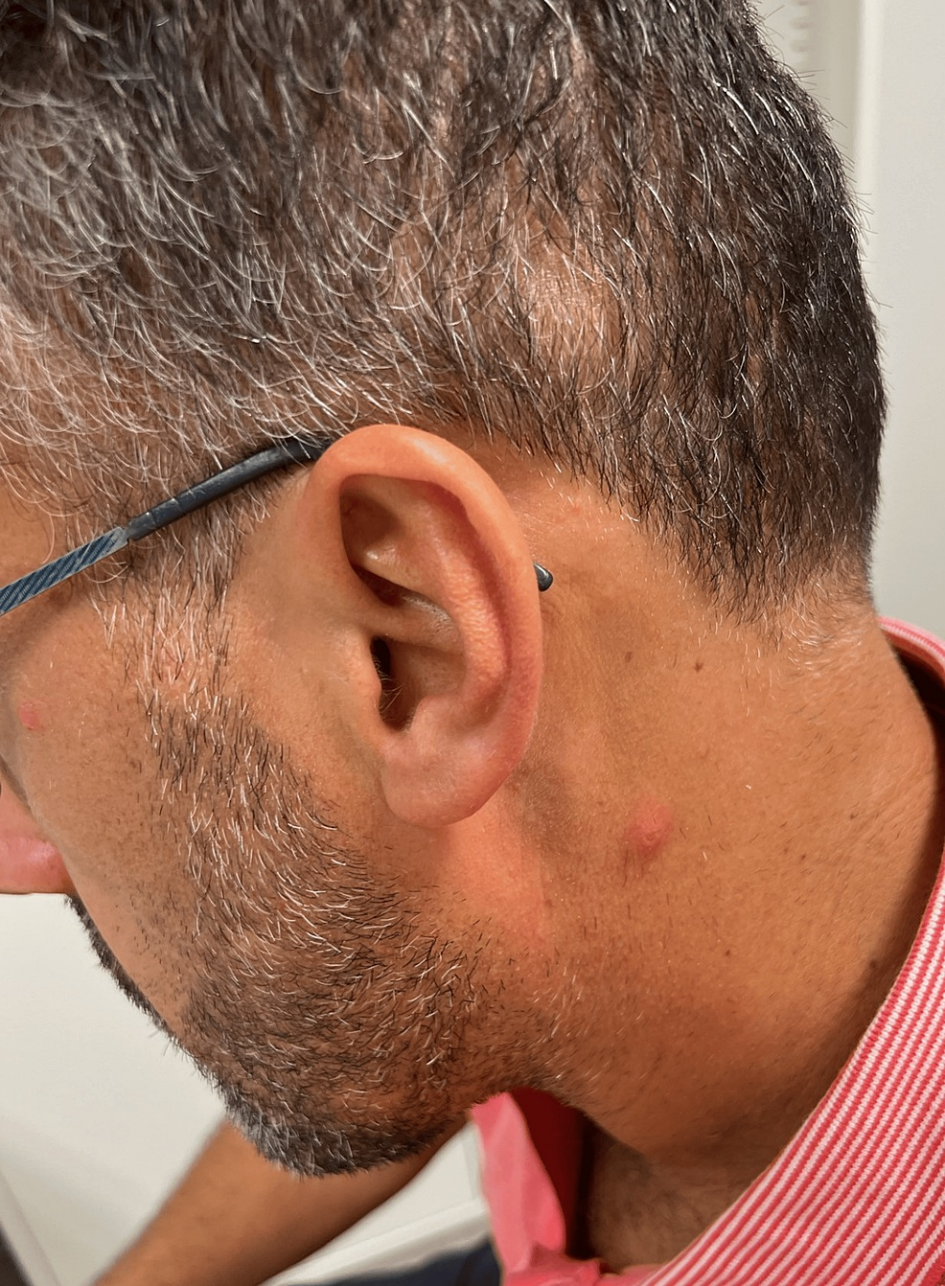
Papular lesion on the face and ulcerated lesion on the neck.

**Figure 2:**
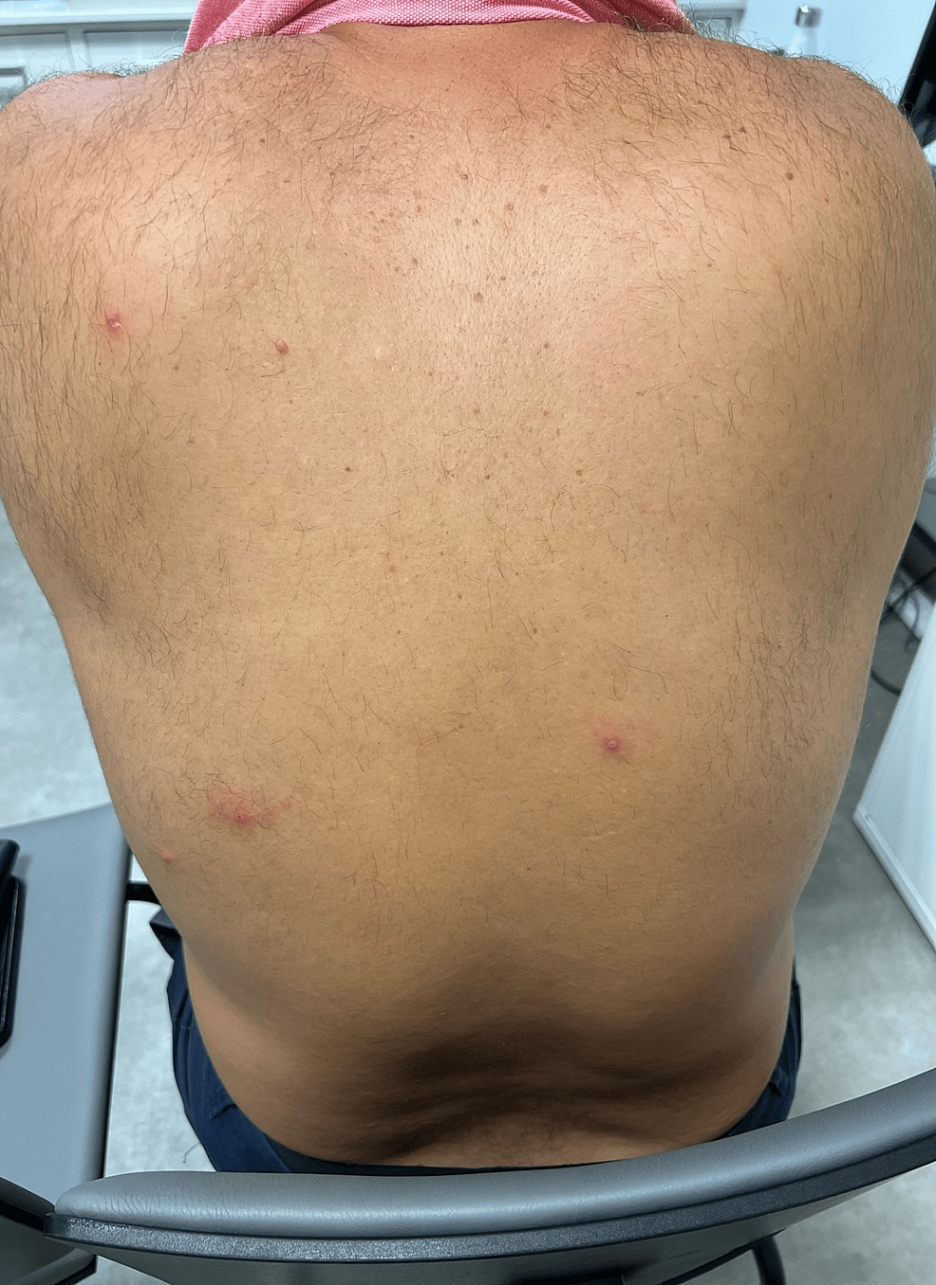
Lesions at different stages on the trunk.

**Figure 3:**
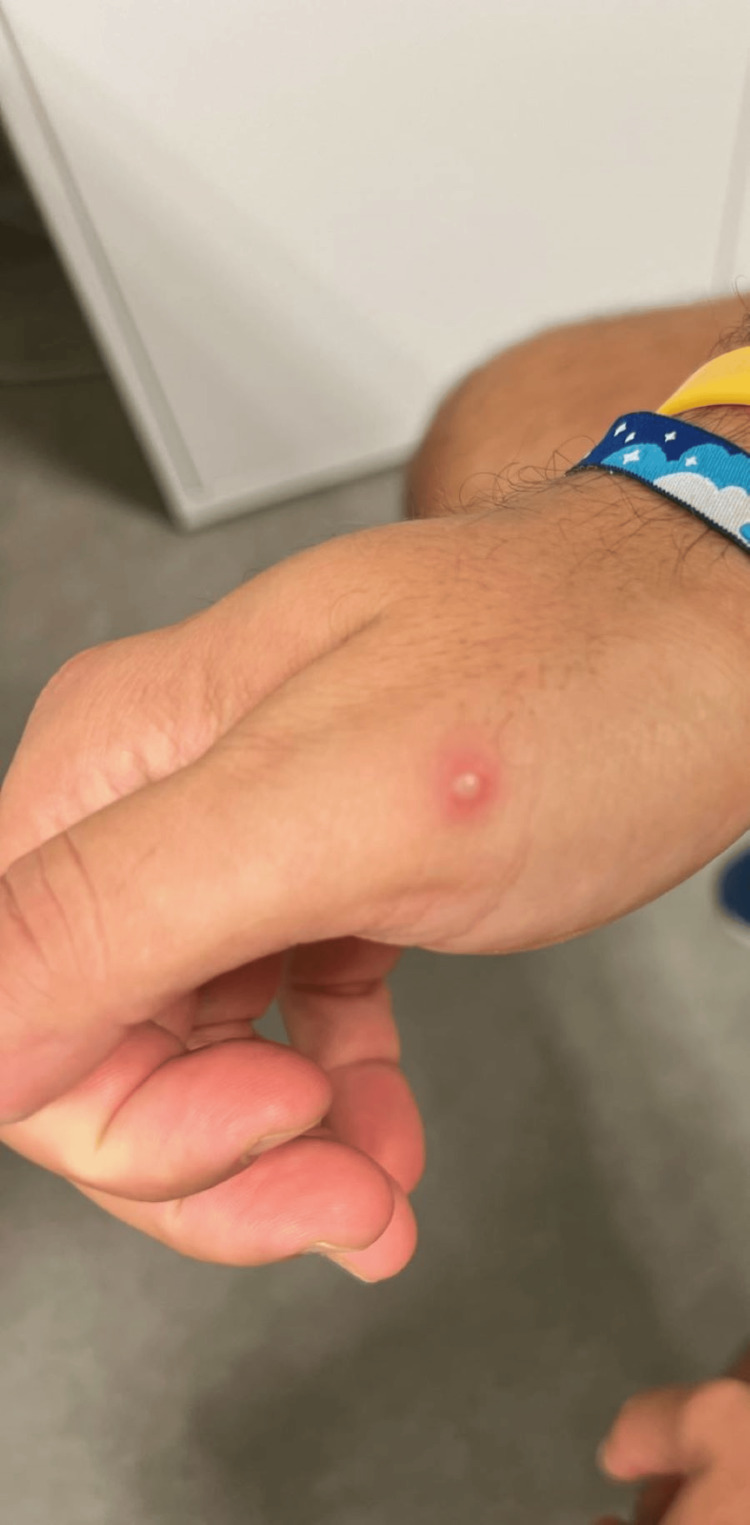
Pustular lesion on the right hand.

On physical examination, findings included lesions at different stages (painful pustules, ulcers, and scabs) on the face, scalp, trunk, and limbs (Figures 1-3). Bilateral painful inguinal lymph nodes were also noted.

The patient was referred to the hospital's emergency department as it was the only available option for collecting samples. Blood tests were performed, including a complete blood count and biochemistry, which revealed no significant abnormalities. Tests for HIV, hepatitis C virus (HCV), and hepatitis B virus (HBV) antibodies were negative. The venereal disease research laboratory (VDRL) test was negative, but the treponemal test (EIA) was positive. Swab samples were collected from the oropharyngeal and vesicular regions for MPXV DNA detection through molecular biology techniques and were sent to a national reference center. All samples tested positive for MPXV infection, although the subtype could not be determined. The patient complied with home isolation and was prescribed supportive treatment, which included oral hydration, antihistamine, and paracetamol as needed. Education on transmission prevention measures was provided. Upon reassessment after seven days, two lesions persisted, leading to continued isolation until resolution. Follow-up with an Infectious Disease specialist in one month, along with repeat viral marker and syphilis tests, was advised.

## Discussion

This report of MPXV infection is remarkable as it represents one of the first documented cases of the disease in a region where its presence, although previously observed, had few to no active or suspected hosts at the moment. MPXV can be transmitted in two ways: from animals to humans and from humans to humans, primarily through contact with infected tissues and fluids. Although human-to-human transmission is less common, any close interaction with an infected person can facilitate it, with contact with respiratory secretions being the most typical route [8-11]. The suspicion of MPXV disease should be considered in light of our patient's recent travel history to Crete, Greece. Despite the patient's denial of direct contact with suspected cases or symptomatic individuals, the possibility of exposure during layovers and crowded events highlights the need for a thorough investigation into prevalent infectious agents when visiting countries with active local transmission [9,10]. The MPXV, similar to smallpox, initiates infection through oropharyngeal or respiratory exposure. After inoculation, the virus primarily replicates at local lymph nodes and then spreads throughout the body, reaching distant organs and other lymph nodes. This widespread viral replication defines the incubation period as lasting seven to 14 days, possibly extending to 21 days, marked by a lack of clinical symptoms and considered non-infectious [5,9]. MPXV infection typically begins with a brief, nonspecific prodromal phase featuring symptoms like fever, myalgia, and lymphadenopathy, lasting around three days. Often mistaken for the common cold, this initial period makes individuals infectious. Notably, the recent MPXV outbreak has revealed cases with mild or unrecognized prodromes, leading to contagious individuals without awareness [8,9]. These appear to align with the patient's symptoms under consideration.

The distinction from other infections, such as smallpox and chickenpox, based solely on clinical presentation can be challenging. However, certain characteristics suggest MPXV infection: smallpox exhibits more severe manifestations and evolves as a monomorphic eruption (vesicles or pustules), unlike MPXV, which presents polymorphically [5,6]; chickenpox lesions are smaller and more superficial when compared to MPXV lesions, distributed on the trunk rather than the limbs [6]; the lymphadenopathy observed in the neck, axilla, or inguinal area, distinguishes MPXV from smallpox and chickenpox, where such involvement is not typically observed [8]. Rapid diagnosis is essential to halt the spread of the disease; however, clinical presentation alone is insufficient for a definitive diagnosis of MPXV. Samples must be collected from cutaneous exudate, vesicular or crusted lesions, and nasopharyngeal/oropharyngeal secretions. Detection of MPXV DNA through real-time polymerase chain reaction (PCR) is the preferred laboratory method due to its sensitivity, with diagnosis confirmed by isolating the virus [9,12]. In the described case, samples of cutaneous vesicle exudates and oropharyngeal were collected, and a search for MPXV DNA was performed using PCR with a positive result. A second confirmatory PCR test was also conducted at the national reference laboratory, the National Institute of Health Dr Ricardo Jorge (INSA), following the established protocol in Portugal [13]. Currently, no targeted treatment exists for MPXV, and although there are suggested interventions, many derived from experience with smallpox, the cornerstone for managing the viral infection is supportive and symptomatic therapy, along with monitoring possible complications [8-10,14,15]. Another focus of patient management involves preventive measures to control spread, including avoiding close contact with others, refraining from sharing objects and common areas (for example, avoiding sharing the same bathroom), frequent handwashing, and avoiding contact with animals, including pets [8,14]. These measures were recommended to the concerned patient and advised to be maintained until crusted lesions have fully healed. After this point, the individual is no longer considered contagious [9,16].

## Conclusions

This case emphasizes the importance of awareness within primary healthcare facilities regarding the potential for MPXV infection, even in regions lacking active or suspected infectious cases. A comprehensive evaluation of recent travel history to areas with confirmed cases proves to be crucial in raising clinical suspicion. The distinctive clinical manifestation of disseminated centrifugal vesiculopustular rash, exhibiting lesions in diverse stages and concurrent lymphadenopathy, strongly supports the clinical suspicion of MPX. PCR-based DNA detection remains the preferred diagnostic modality, corroborated by viral identification in oropharyngeal secretions or lesion exudate. Therapeutic management focuses on supportive care, monitoring, intervention for potential complications, and enforcement of preventive contact measures to control the outbreak of MPXV infection.
